# Treatment Preferences in Patients With Insomnia and Medical Comorbidity: Associated Factors and Impact on Treatment‐Outcome

**DOI:** 10.1111/jsr.70115

**Published:** 2025-06-30

**Authors:** Nynke Rauwerda, Irene Pot, Annemarie Braamse, Annemieke van Straten, Pythia van Nieuwkerk, Myrthe Boss, Marian Rikkert, Hans Knoop

**Affiliations:** ^1^ Department of Medical Psychology Amsterdam University Medical Center, University of Amsterdam, Amsterdam Public Health Research Institute Amsterdam the Netherlands; ^2^ Department of Medical Psychology Hospital Gelderse Vallei Ede the Netherlands; ^3^ Department of Clinical Psychology & Amsterdam Public Health Research Institute VU University Amsterdam the Netherlands; ^4^ Department of Neurology Hospital Gelderse Vallei Ede the Netherlands; ^5^ Department of Medical Psychology Hospital Rivierenland Tiel the Netherlands

**Keywords:** amitriptyline, CBT‐I, cognitive behavioural therapy for insomnia, medical conditions

## Abstract

Insomnia is common in patients with medical comorbidity. First‐line treatment for insomnia is cognitive behavioural therapy for insomnia (CBT‐I). However, some patients with medical comorbidities prefer pharmacological treatment. This study aimed to (1) identify factors influencing treatment preference in these patients, and (2) assess how aligning treatment with patient preferences impacts outcomes for CBT‐I and low‐dose Amitriptyline. This study was part of a non‐inferiority randomised controlled trial. The study involved 187 participants who were randomly assigned to either CBT‐I or Amitriptyline for 12 weeks and 54 participants who refused to participate in the randomised controlled trial. Treatment preferences were assessed at baseline using the Treatment Perception and Preferences (TPP) questionnaire and insomnia severity was assessed before and after treatment with the Insomnia Severity Index (ISI). Multiple and linear regression analyses (*p* < 0.001, adj. *R* ^2^ = 0.06) revealed that higher age and attributing insomnia to psychological causes predicted a stronger preference for CBT‐I while severe insomnia predicted a stronger preference for Amitriptyline. There were no differences in treatment‐outcome between amitriptyline and CBT‐I for those without a treatment preference, neither for those receiving their treatment preference. However, for patients not receiving their treatment preference, amitriptyline performed significantly worse than CBT‐I (*M* = 13.72 (1.72) vs. *M* = 9.83 (0.80), *p* = 0.045). In conclusion, age, attributing insomnia to psychological causes and insomnia severity predict treatment preference in patients with medical comorbidity. Our findings suggest that when treatment does not align with a patient's preference, CBT‐I results in a better treatment‐outcome than medication.

**Trial Registration:** Dutch Trial Register: NTR NL7971

## 1 Introduction

The prevalence of insomnia among patients with medical conditions is high, with reports of up to 66% (Budhiraja et al. 2011; Katz and McHorney 1998; Taylor et al. 2007). Insomnia adversely affects the course and prognosis of medical conditions (Dikeos and Georgantopoulos 2011). Treating insomnia in these patients is important, as improving sleep can enhance both physical health and daily functioning (Wu et al. 2015).

The treatment of insomnia can be divided into pharmacological and non‐pharmacological interventions. Guidelines recommend Cognitive Behavioural Therapy for Insomnia (CBT‐I) as the first‐line treatment (Riemann et al. 2023). CBT‐I has been proven effective in reducing insomnia, even in patients with medical comorbidities (Okajima and Inoue 2018; Smith et al. 2005; Wu et al. 2015). In addition to improving sleep, CBT‐I can also reduce symptoms associated with other health conditions, such as pain (Okajima and Inoue 2018). However, not all patients are willing to participate in CBT‐I, nor do they always have access to it (Buenaver et al. 2019; Koffel et al. 2018). As a result, pharmacological treatments, such as benzodiazepine receptor agonists or off‐label antidepressants like amitriptyline, are commonly used despite limited evidence of their long‐term efficacy (Everitt et al. 2018; Riemann and Perlis 2009).

An important factor in selecting the most appropriate treatment is patient preference. Although clinicians often assume that patients prefer medication, research shows that many patients, including those with medical comorbidities, actually favour psychological treatments like CBT‐I over pharmacological options (Harvey et al. 2013; Morin et al. 1992; Omvik et al. 2010). In patients without comorbidities, various factors like previous medication use, insomnia severity, fatigue levels, and beliefs about sleep can influence treatment preferences (Bluestein et al. 2011; Cheung et al. 2018; Perez et al. 2022; Sidani, Miranda, Epstein, Bootzin, et al. 2009; Vincent and Lionberg 2001). However, it is not clear whether these same factors influence treatment preferences in patients with medical comorbidities. For this population, additional factors such as beliefs about the psychological or somatic causes of their insomnia (Shaffer et al. 2020) or pain‐related factors (Ravyts et al. 2022) may also play a significant role in treatment preferences. Understanding factors associated with preferences, may facilitate targeted education and shared decision making with regard to insomnia treatment.

Misalignment between the treatment offered and the patient's preference can negatively affect adherence, which may lead to poorer treatment outcomes (Cheung et al. 2016; Sidani, Miranda, Epstein, and Fox 2009). If patients feel that their treatment is not aligned with their preferences, they may be less motivated to engage, reducing the likelihood of success. The impact of aligning treatment with treatment preference can be even more important in patients with medical comorbidity because CBT‐I requires substantial patient engagement, which can be challenging for individuals with chronic health issues. In contrast, pharmacological treatments, while easier to adhere to, carry risks such as dependence and adverse side effects, which can be especially problematic for patients already managing multiple health conditions (Cheung et al. 2019).

This study aimed to determine factors that predict treatment preferences (CBT‐I vs. pharmacological treatment) in patients with insomnia and medical comorbidities, focusing on sociodemographic characteristics, cognitive‐behavioural factors (such as beliefs about causes of insomnia), and clinical variables. A second aim of the study was to examine how aligning treatment with patient preferences impacts treatment outcome.

## 2 Materials and Methods

### 2.1 Study Design and Participants

The data of this study were collected within the context of the TIMELAPSE study, registered in the Dutch trial register (NTR NL7971) and approved by the Amsterdam University Medical Centre Research Ethics Committee (reference 2019_101) and the ethics committees of the participating hospitals. The TIMELAPSE study was a multicenter non‐inferiority randomised controlled trial that investigated whether low dosage (10–20 mg nightly) Amitriptyline (AM) is a non‐inferior treatment compared to CBT‐I for patients with insomnia disorder and medical comorbidity (Rauwerda et al. 2021). Results showed that AM is non‐inferior to CBT‐I in reducing insomnia severity in patients with medical comorbidities, but has more side effects and its effect on insomnia may diminish after tapering (Rauwerda et al. forthcoming). Eligible patients were aged between 18 and 85 years, met the DSM‐5 criteria for insomnia (American Psychiatric Association 2013) and scored ≥ 10 on the Insomnia Severity Index (Morin et al. 2011). Further, they had a long‐term medical condition and/or persistent physical complaints (> 3 months) that required medical attention, and caused dysfunction, discomfort or social problems. TIMELAPSE study‐related exclusion criteria are described in Table 1. Patients with a severe psychiatric disorder which was not in remission or not adequately treated were excluded. Psychiatric conditions were not seen as medical comorbidity. Current use of benzodiazepines was not an exclusion criterion.

**TABLE 1:** In‐ and exclusion criteria.

Inclusion criteria	Exclusion criteria
18–85 years Insomnia disorder according to the criteria of the DSM‐5 Score of ≥ 10 on the Insomnia Severity Index Long term medical condition	*Study related exclusion criteria*: Habitual night shifts Untreated sleep related breathing disorder Wish to continue over‐the‐counter sleep aids Off‐label amitriptyline for insomnia in past year Epilepsy, dementia, history of delirium Terminal illness Severe psychiatric disorder Substance abuse/addiction (benzodiazepine excluded)
	*Potential drug–drug interactions for amitriptyline*: Use of psychopharmaceuticals (other than benzodiazepine) or antimycotics
	*Contra‐indications for amitriptyline*: Allergy for amitriptyline Cardiac disorders Severe renal insufficiency or liver dysfunction Pregnancy, lactation or pregnancy wish Ocular hypertension/glaucoma

Since low‐dose AM was only prescribed in the TIMELAPSE study, participants in this study may have had a preference for AM. Participation in the TIMELAPSE study provided them with the opportunity to receive AM, whereas in routine clinical care, they would typically be offered a non‐pharmacological treatment. In contrast, individuals who preferred CBT‐I might have declined participation in TIMELAPSE as standard clinical care provided more opportunities to receive CBT‐I than participating in the TIMELAPSE study. Therefore, to gather a more representative sample for the first aim of the study (identifying factors influencing treatment preference in the total group of patients with insomnia and medical co‐morbidity), we also added treatment preference data from 54 non‐TIMELAPSE patients. These patients declined TIMELAPSE but agreed to complete questionnaires before starting treatment outside the study (e.g., behavioural therapy or other pharmacological treatment). The decision to expand the sample was made post hoc upon observing that the majority of participants in the initial study had a preference for medication. In this way we wanted to get a more representative sample of the broader population of individuals with insomnia and medical comorbidities, which was the goal of the study.

There are no treatment outcome data for this subgroup and no additional ethical approval was required to include this subgroup for this part of the study (Amsterdam UMC, reference W20_366 # 20.409).

### 2.2 Procedures

Patients were recruited from outpatient clinics of Neurology and Medical Psychology departments at four general hospitals in the Netherlands between September 2019 and April 2024. After providing informed consent, all participants (TIMELAPSE and non‐TIMELAPSE) completed an online assessment, including insomnia treatment preferences. Only TIMELAPSE participants were randomised into two groups: (1) group CBT‐I or (2) low‐dose AM, in blocks of 2 or 4 (1:1 ratio). Elements of the CBT‐I were: psycho‐education, sleep hygiene, relaxation, stimulus control and sleep restriction, cognitive interventions and relapse prevention. Non‐TIMELAPSE participants completed the assessment before starting treatment outside the TIMELAPSE study. Data were stored online in a structured database. TIMELAPSE participants completed self‐report measures 12 weeks after start of treatment. The latter was the primary endpoint of the study. For a detailed description of the study we refer to the published protocol (Rauwerda et al. 2021).

### 2.3 Measures

#### 2.3.1 Treatment Preference

The Treatment Perception and Preferences (TPP) questionnaire assessed insomnia treatment acceptability and preferences (Sidani et al. 2018). Translated into Dutch and back‐translated, the TPP described CBT‐I or low‐dose AM, including their goals, activities, delivery, benefits, and risks, see Appendix A. To control for order biases, the order of treatment descriptions was alternated. Because treatment preference for pharmacological and non‐pharmacological treatment are often not mutually exclusive (Bluestein et al. 2011) and the value of single item assessment is limited (Cheung et al. 2016), treatment preference as measured with the TPP was operationalised in two different ways. (1) Treatment acceptability was assessed as a continuous variable; participants rated each treatment on nine attributes (e.g., appropriateness, effectiveness, risks, convenience) using a 5‐point scale. The item on risks was recoded so that higher ratings indicated greater acceptability. In this study, internal consistency of the CBT‐I and AM subscales were good and excellent, respectively (Cronbach's *α* = 0.88 and 0.90). Mean scores (TPP‐acceptability) for each treatment was computed. A difference score (TPP‐acceptability AM minus TPP‐acceptability CBT‐I) was computed (TPP‐Δ acceptability), with a score > 0 presenting higher acceptability for AM, while < 0 for CBT‐I. Previous research on the TPP indicated that patients who preferred one treatment also rated it as more acceptable (Sidani et al. 2018). Therefore, this TPP‐Δ acceptability score serves as an indirect assessment of treatment preference. (2) Treatment preferences (TPP‐preference) were assessed directly as well with two categorical questions: (1) “Do you have a preference (yes/no)?”, (2) “Which treatment do you prefer (AM/CBT‐I)?”

#### 2.3.2 Sociodemographic Predictors of Treatment Preference

The survey included questions regarding sex, age, marital status (partnered, single, and other), employment status (employment, [temporarily] medically unfit for work, unemployed, studying, and retired), and educational level (high: university or higher vocational education, middle: secondary education, and low: primary education).

#### 2.3.3 Clinical Predictors of Treatment Preference

Participants' medical conditions and current use of hypnotics were assessed by the neurologist.

Depression was assessed with the 7‐item subscale depression of the Hospital Anxiety and Depression Scale (HADS) (Bjelland et al. 2002). Scoring ≥ 8 is indicative for moderate symptoms of depression (Razavi et al. 1990). The psychometric properties of the Dutch version of the HADS are adequate (Spinhoven et al. 1997). In this study, internal consistency was good (Cronbach's *α* = 0.84).

Fatigue was assessed with the 8‐item subscale Fatigue severity of the Checklist Individual Strength (CIS‐fat). Items are scored on a seven‐point scale (1‐7) (Dikeos and Georgantopoulos 2011; Katz and McHorney 1998; Okajima and Inoue 2018; Riemann et al. 2023; Taylor et al. 2007; Wu et al. 2015). A score of 35 or higher indicates severe fatigue. The CIS‐fat is reliable and valid (Worm‐Smeitink et al. 2017). In this study, internal consistency was good (Cronbach's *α* = 0.87).

Physical functioning was assessed with the 10‐item physical functioning subscale of the Short Form‐36 item Health Survey (SF‐36). Scores were transformed and range from 0 to 100, with higher scores indicating better physical functioning. The physical functioning subscale of the SF‐36 is valid and reliable (Aaronson et al. 1998). In this study, internal consistency was excellent (Cronbach's *α* = 0.91).

Interference of pain on daytime functioning was assessed with the Subscale interference of bodily pain of the SF‐36. The item interference of pain ranges from 0 to 50. Higher scores indicate less interference (Aaronson et al. 1998).

#### 2.3.4 Cognitive‐Behavioural Predictors

Dysfunctional beliefs and attitudes about sleep were assessed with the 16‐item Dysfunctional Belief and Attitudes about Sleep scale (DBAS‐16), which has good internal consistency and test–retest reliability (Morin et al. 2007). In this study, internal consistency was good (Cronbach's *α* = 0.87). Items of the DBAS‐16 are rated on an 11‐point Likert scale (range 0–10). Sum scores were averaged so they ranged from 0 (no) to 10 (severe dysfunctional beliefs and attitudes about sleep). A score of ≥ 3.8 is indicative of dysfunctional sleep beliefs and attitudes about sleep (Carney et al. 2010).

Causal attributions of insomnia were assessed using 12 items of the Causal Attributions of my Insomnia questionnaire (CAM‐I) (Harvey et al. 2013), which were also translated into Dutch using a forward‐backward method. The items reflect 12 psychological and somatic/biological domains that are considered to contribute to insomnia. Because of the medical comorbidity in our sample, we added three new domains; medical illness, bodily sensations and side effects of pharmacological treatment for medical illness. Participants were asked the following question: “How likely do you think it is that the following factors contributed to your insomnia?” The domains were rated on a 7‐point Likert scale. A confirmatory factor analysis was conducted to examine the presence of two components, which we hypothesized to be predictors of treatment preference: psychological and somatic/biological. There were two components with an eigenvalue ≥ 1. One was a reliable “psychological” component (Cronbach's *α* = 0.82), encompassing the domains of sleep‐related thoughts, bodily arousal, cognitive arousal, sleep‐related emotions, and general emotions. However, the reliability of the “somatic/biological” component was questionable (Cronbach's *α* = 0.62).

#### 2.3.5 Insomnia Severity

Insomnia severity was measured with the 7‐item Insomnia Severity Index (ISI) (Morin et al. 2011). The items are rated on a 5‐point Likert scale (range 0–4). The ISI is a valid and reliable instrument (Bastien et al. 2001). In this study, Cronbach's *α* was 0.66, which is acceptable. Insomnia severity was measured before treatment as a potential predictor of treatment preference, and again after 12 weeks of treatment to assess treatment outcomes. A score of ≥ 10 was used to define clinical insomnia (Buysse et al. 2006).

### 2.4 Statistical Analysis

All statistical analyses were performed using IBM SPSS Statistics (version 28). Descriptive statistics were used to summarise participants' sociodemographic, clinical, and cognitive‐behavioural characteristics. Differences between TIMELAPSE and non‐TIMELAPSE participants were compared using *χ* ^2^ tests or independent samples *t* tests, depending on the distribution of the data. Differences within groups were compared by using paired *t* tests.

To investigate the relationship between treatment preference and potential predictors, multiple regression analysis was conducted on the total sample. The dependent variable was the difference in mean acceptability scores between low‐dose AM and CBT‐I (TPP‐Δ acceptability). Independent variables included age, sex, educational level, current use of sleep medication, insomnia severity, depression, fatigue severity, physical functioning, interference of pain on daytime functioning, dysfunctional beliefs and attitudes about sleep, and causal attributions of insomnia (psychological and somatic/biological). Univariate regression analyses were first performed to preselect predictors. Following Hosmer et al. (2013) all predictors with a *p* < 0.25 were included in our multivariate regression analysis, followed by backward elimination. In a sensitivity analysis, the multivariate regression model was repeated for the TIMELAPSE sample alone.

Our sample size was based on the required sample size for the primary outcome of the TIMELAPSE study (non‐inferiority trial AM vs. CBT‐I), and additional participants were included who had declined participation in the TIMELAPSE study. An a priori sample size calculation was conducted to determine if the number of participants was adequate for the present sub‐study (investigating factors associated with treatment preferences) using G*Power (Faul et al. 2009) for linear multiple regression with 12 predictors. This calculation indicated that a minimum sample size of 127 was needed to achieve a power of 0.80 and detect at least a moderate effect size (*f* ^2^ = 0.15) at an *α* level of 0.05.

Further analyses were conducted to explore the relationship between treatment preference and treatment outcomes. The TIMELAPSE participants were categorised into three groups based on their treatment preference (TPP‐preference: no preference, AM or CBT‐I) and whether they received their preferred treatment: (1) no treatment preference, (2) received preferred treatment, and (3) not received preferred treatment. In each category, a one‐way ANCOVA was performed to assess the difference between both treatments (CBT‐I vs. AM) in treatment outcome (insomnia severity), as measured by the Insomnia Severity Index (ISI) at 12 weeks treatment. Baseline ISI score was included as a covariate in the analysis. Only participants who completed the ISI post‐treatment were included. Additionally, linear regression was performed in the TIMELAPSE sample to examine the relationship between the strength of treatment acceptability (as a continuous variable, represented by TPP‐Δ acceptability) and ISI scores at 12 weeks. The regression model included the TPP‐Δ acceptability, baseline ISI score, and treatment group as independent variables, with ISI scores at 12 weeks as the dependent variable.

## 3 Results

### 3.1 Participant Characteristics

The total study sample consisted of 241 participants, 187 participants of the TIMELAPSE study and 54 non‐TIMELAPSE participants. The descriptive statistics are presented in Table 2. The age of the total sample ranged from 20 to 80 years, with an average of 54 years. The majority of participants were female (62%) and 30% used B(Z)RA's before the start of the study (see Table 2). Forty‐four percent had more than one medical condition. Appendix B presents the medical conditions of the sample, categorised in main ICD‐11 categories. The mean level of insomnia severity was 17.3. Many participants (*N* = 95, 39%) reported elevated levels of depressive symptoms on the HADS, and most severe fatigue on the CIS‐fat (*N* = 186, 77%) and sleep‐related dysfunctional beliefs and attitudes (*N* = 202, 84%), see Table 2. The mean level of psychological causal attributions was 17.2 (7.3) and 14.9 (6.1) for somatic/biological causal attributions.

**TABLE 2:** Sociodemographic, clinical and cognitive behavioural characteristics for the total sample, TIMELAPSE participants and non‐TIMELAPSE participants.

	Total sample (*N* = 241)	TIMELAPSE participants (*N* = 187)	Non‐TIMELAPSE participants (*N* = 54)	*p*
Sociodemographic characteristics				
Age, mean (±sd)	54.0 (12.1)	52.7 (12.3)	58.2 (10.3)	0.003*
Male, *N* (%)	91 (38%)	76 (41%)	15 (28%)	0.11
Marital status: partner, *N* (%)	183 (76%)	144 (77%)	39 (71%)	0.47
Educational level, *N* (%)				
Low	31 (13%)	25 (14%)	6 (11%)	0.50
Middle	99 (41%)	73 (39%)	26 (47%)	
High	110 (46%)	88 (47%)	22 (40%)	
Employment status: employment, *N* (%)	141 (59%)	112 (60%)	29 (54%)	0.44
Clinical characteristics				
1 medical condition, *N* (%)	134 (56%)	100 (54%)	34 (63%)	0.28
≥ 2 medical conditions, *N* (%)	106 (44%)	87 (46%)	20 (37%)	
Current use of B(Z)RA's^a^, *N* (%)	73 (30%)	56 (30%)	17 (31%)	0.83
Insomnia severity, mean (±sd)	17.3 (4.0)	17.9 (3.5)	15.3 (4.9)	< 0.001*
Depression, mean (±sd)	6.5 (4.2)	6.7 (4.3)	6.0 (3.7)	0.30
Fatigue severity, mean (±sd)	40.8 (9.8)	41.9 (9.4)	37.2 (10.1)	0.004*
Physical functioning, mean (±sd)	73.0 (22.7)	71.7 (23.1)	77.5 (20.7)	0.08
Interference of pain, mean (±sd)	36.3 (13.2)	35.6 (13.8)	38.5 (10.7)	0.15
Cognitive behavioural characteristics				
Dysfunctional beliefs and attitudes about sleep, mean (±sd)	5.2 (1.6)	5.4 (1.4)	4.7 (1.9)	0.003*
Causal attributions of insomnia				
Psychological, mean (±sd)	17.2 (7.3)	17.3 (7.4)	16.9 (7.1)	0.69
Somatic/biological, mean (±sd)	14.9 (6.1)	14.9 (6.1)	14.6 (6.3)	0.79

^a^ Zopiclone, Zolpidem, Oxazepam, Diazepam, Lorazepam, Lormetazepam, Temazepam, and Midazolam.

* *p* < 0.05.

#### 3.1.1 Differences Between TIMELAPSE and Non‐TIMELAPSE Participants at Baseline

Compared to the group of 54 non‐TIMELAPSE participants, TIMELAPSE participants were significantly younger, reported more severe insomnia and more dysfunctional beliefs and attitudes about sleep.

### 3.2 Treatment Preference

As shown in Table 3, 68% of the participants in the total sample had a treatment preference. Of these participants, 68% preferred low‐dose AM, and 32% preferred CBT‐I. When a preference was present, TIMELAPSE participants preferred low‐dose AM (*N* = 103, 80%) above CBT‐I (*N* = 25, 20%) and non‐TIMELAPSE participants preferred CBT‐I (*N* = 27, 75%) above low‐dose AM (*N* = 9, 25%). This group difference was significant (*p* < 0.001). TIMELAPSE participants reported higher acceptability of both low‐dose AM and CBT‐I than non‐TIMELAPSE participants (both *p* < 0.001).

**TABLE 3:** Insomnia treatment preference and treatment acceptability for the total sample, TIMELAPSE participants and non‐TIMELAPSE participants.

	Total sample (*N* = 241)	TIMELAPSE participants (*N* = 187)	Non‐TIMELAPSE participants (*N* = 54)	*p*
Treatment preference (TPP‐preference), *n* (%)				
CBT‐I	52 (22%)	25 (13%)	27 (50%)	< 0.001*
Low‐dose AM	112 (47%)	103 (56%)	9 (17%)	
No treatment preference	77 (32%)	59 (31%)	18 (33%)	
Treatment acceptability (TPP‐acceptability), mean (±sd)				
CBT‐I	1.87 (0.95)	1.99 (0.61)	1.48 (0.79)	< 0.001*
Low‐dose AM	2.11 (0.68)	2.45 (0.70)	0.94 (0.79)	< 0.001*
Δ Treatment acceptability (TPP‐Δ acceptability), mean (±sd)				
	0.24 (1.00)	0.46 (0.88)	−0.57 (1.01)	< 0.001*

Abbreviations: TPP: treatment perception and preference questionnaire; TPP‐acceptability: acceptability subscale of the TPP; higher score indicates greater acceptability; TPP‐preference: preference subscale of the TPP; TPP‐Δ acceptability: difference score of TPP‐acceptability AM‐CBT‐I, a positive score indicates higher acceptability in favour of AM.

* *p* < 0.05.

Exploratory analysis showed that in the total sample (TIMELAPSE and non‐TIMELAPSE participants) low‐dose AM was perceived as more effective in the short term (Δ 0.73, *p* < 0.001), in reducing fatigue (Δ 0.20, *p* = 0.01) and improving daytime functioning (Δ 0.15, *p* = 0.04) and easier to apply (Δ 1.0, *p* < 0.001) than CBT‐I. Risks and side effects of low‐dose AM were perceived as more severe (Δ −0.67, *p* < 0.001) than CBT‐I.

### 3.3 Predictors of Insomnia Treatment Preference

Age, insomnia severity, dysfunctional beliefs and attitudes about sleep, attribution of psychological causes to insomnia, physical functioning, current use of sleep medication, and fatigue severity were selected as potential predictors for the multivariable regression analysis because their *p* values were less than 0.25 in the univariate regression analysis. In contrast, the variables sex, educational level, depression, the impact of bodily pain on daytime functioning, and attribution of somatic causes to insomnia were excluded, as their *p* values exceeded 0.25 in the univariate regression analyses (see Table 4).

**TABLE 4:** Univariate regression analyses of potential factors associated with treatment preference.

	Stand. *β*	*p*	Adj. *R* ^2^
Sociodemographic factors			
Age	−0.14	0.03*	0.02
Sex	0.03	0.67	−0.003
Educational level	−0.04	0.59	−0.003
Clinical factors			
Current use of sleep medication	0.09	0.17*	0.004
Insomnia severity	0.19	0.003*	0.03
Depression	0.03	0.60	−0.003
Fatigue severity	0.13	0.05*	0.01
Physical functioning	−0.11	0.09*	0.01
Interference of pain on daytime functioning	−0.07	0.28	0.001
Cognitive behavioural factors			
Dysfunctional beliefs and attitudes about sleep	0.16	0.02*	0.02
Causal attributions of insomnia			
Psychological	−0.09	0.18*	0.003
Somatic/biological	−0.001	0.99	−0.004

* *p* < 0.25.

In the final model treatment preference was predicted by age, attributing insomnia to psychological causes and insomnia severity (see Table 5; *p* < 0.001, adj. *R* ^2^ = 0.07). Higher age and attributing psychological causes to insomnia predicted a stronger preference for CBT‐I and higher insomnia severity predicted a stronger preference for AM. When repeating the multivariate regression analysis in the TIMELAPSE sample, we found no significant predictors of treatment preference (see Data S1 for more details).

**TABLE 5:** Multivariate regression analyses of factors associated with insomnia treatment preference.

Model^a^	I		II		III		IV		V	
Predictor	Stand. *Β*	*p*	Stand. *Β*	*p*	Stand. *Β*	*p*	Stand. *Β*	*p*	Stand. *Β*	*p*
Age	−0.17	0.01*	−0.17	0.01*	−0.15	0.02*	−0.14	0.03*	−0.15	0.02*
Insomnia severity	0.15	0.06	0.15	0.15	0.13	0.08	0.14	0.05*	0.21	0.001*
Psychological attribution	−0.18	0.01*	−0.18	0.01	−0.17	0.02*	−0.17	0.01*	−0.13	0.04*
Dysfunctional beliefs and attitudes about sleep	0.14	0.09	0.16	0.05	0.13	0.09	0.14	0.08		
Physical functioning	−0.10	0.19	−0.10	0.20	−0.06	0.34				
Fatigue severity	−0.08	0.35	−0.09	0.32						
Current use of sleep medication	0.05	0.42								
Adj. *R* ^2^	0.07	0.002	0.07	0.001*	0.07	< 0.001*	0.07	< 0.001*	0.06	< 0.001*
*R* ^2^	0.09		0.09		0.09		0.08		0.07	
*N*	237		238		238		239		239	

^a^ Model I included all candidate variables (*p* < 0.25 in univariate regression analyses). In the subsequent models, non‐significant variables (*p* > 0.05) were sequentially removed based on backward selection criteria until the final model (V) retained only significant predictors.

* *p* < 0.05.

### 3.4 Aligning Preference to Treatment

**FIGURE 1:**
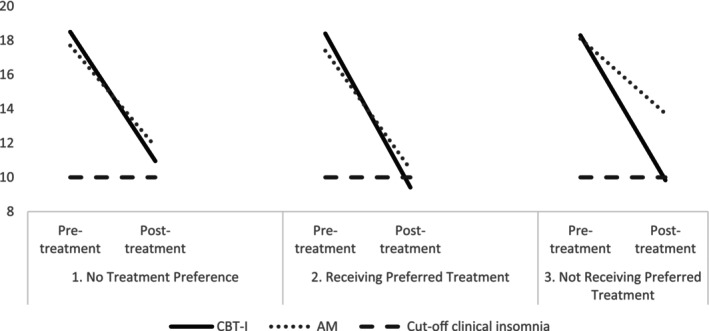
Insomnia severity pre‐and post‐treatment (controlled for baseline insomnia severity) of CBT‐I and AM as measured with the Insomnia Severity Index (ISI) in participants (1) with no treatment preference, (2) receiving preferred treatment, and (3) not receiving preferred treatment.

A first analysis investigated the effect of treatment preference (categorical variable TPP‐prereference) on treatment‐outcome (ISI score), when adjusted for baseline ISI‐score. The TIMELAPSE participants were categorised into three groups based on their categorical treatment preference and their received treatment, see Figure 1:

1. *No treatment preference* (*N* = 51): There was no statistically significant difference on the ISI‐scores between the AM and the CBT‐I group at 12 weeks (AM: *N* = 28, *M* = 11.79 (SE = 1.17), CBT‐I: *N* = 23, *M* = 10.95 (SE = 1.29), *p* = 0.63).
2. *Received preferred treatment* (*N* = 64): Similar to the no‐preference group, there was no statistically significant difference in ISI‐scores at 12 weeks between those who received AM and those who received CBT‐I (AM: *N* = 50, *M* = 10.47 (SE = 0.73), CBT‐I: *N* = 14, *M* = 9.41 (SE = 1.38), *p* = 0.50).
3. *Did not receive preferred treatment* (*N* = 57): There was a significant difference in ISI‐scores at 12 weeks between the AM‐group and the CBT‐I‐group. Participants assigned to AM had significantly higher ISI‐scores compared to those assigned to CBT‐I (AM: *N* = 10, *M* = 13.72 (SE = 1.72) vs. CBT‐I: *N* = 47, 9.83 (SE = 0.80), *p* = 0.045).

A second analysis examined the impact of treatment preference using the continuous TPP‐Δ acceptability score in a linear regression. Neither the TPP‐Δ acceptability score (*β* = −0.13, *p* = 0.08) nor treatment group (AM vs. CBT‐I) (*β* = 0.13, *p* = 0.08) significantly predicted ISI scores at week 12; baseline ISI score did (*β* = 0.36, *p* < 0.001).

## 4 Discussion

This is the first study investigating a broad range of sociodemographic, clinical and cognitive‐behavioural predictors of treatment preference (CBT‐I vs. low‐dose AM) and the impact of treatment preference on treatment‐outcome in patients with insomnia disorder and medical comorbidity. We found that in this patient group higher age and attributing insomnia to psychological causes predicted a preference for CBT‐I and higher insomnia severity a preference for AM. Furthermore we found that when treatment did not align with a patient's preference, CBT‐I resulted in better treatment‐outcome than medication.

Higher age predicted a stronger preference for CBT‐I relative to AM. A previous study, mainly focusing on behavioural treatments in primary care, found higher age to be associated with disliking behavioural insomnia treatment (Perez et al. 2022). Elderly patients with medical comorbidities in our sample preferred CBT‐I, maybe due to concerns about side effects and drug interactions from their existing medication. Our finding that attributing insomnia to psychological factors like cognitive arousal and sleep‐related thoughts as causes of insomnia drove a preference for CBT‐I is consistent with previous research on insomnia (Harvey et al. 2013) and other mental disorders (Riedel‐Heller et al. 2005).

Higher insomnia severity predicted a stronger preference for low‐dose AM relative to CBT‐I. Inconsistencies were found in previous studies regarding an association between insomnia severity and preference for medication (Bluestein et al. 2011; Perez et al. 2022). In our study, participants expected low‐dose AM to be more effective in improving sleep in the short term, fatigue, and daytime functioning and easier to use than CBT‐I. This may explain why those with severe insomnia preferred medication. Given their high insomnia burden, they were likely seeking a quick, effective, and easy treatment for both night and daytime symptoms, which they hoped AM could provide.

As Morin et al. (1992), we did not find an association between sex and treatment preference. Unlike previous research, we did not find an association between treatment preference and educational level, current use of sleep medication, physical functioning, depression, fatigue severity, or dysfunctional beliefs and attitudes about sleep (Bluestein et al. 2011; Cheung et al. 2018; Perez et al. 2022; Sidani, Miranda, Epstein, Bootzin, et al. 2009; Vincent and Lionberg 2001). Not replicating these findings might be due to methodological differences. Previous studies did not specifically focus on patients with medical comorbidities. Furthermore, some studies focused only on preferences for non‐pharmacological treatments. In our study, patients with higher interference of pain on daytime functioning or attributing their insomnia to somatic/biological causes did not have a stronger preference for pharmacological treatment, in this case low‐dose AM, although previous studies suggested this association (Ravyts et al. 2022; Shaffer et al. 2020). Not finding this association might be due to the questionable reliability of the somatic component of the CAM‐I. When repeating the multivariate regression analysis in the TIMELAPSE sample, we no longer found significant predictors of treatment preference, likely due to the underrepresentation of patients preferring CBT‐I.

In addition to identifying factors associated with treatment preferences, we aimed to assess how treatment preference influences treatment‐outcome. Our findings revealed no significant difference in insomnia severity at 12 weeks for participants who received their preferred treatment, indicating that both CBT‐I and AM were equally effective. When aligned with patient preferences. Findings were similar in patients with no preference. However, participants who preferred CBT‐I but received AM reported significantly worse outcomes, suggesting that misalignment between patient preference and prescribed treatment can reduce the effectiveness of a pharmacological intervention. While the effectiveness of AM depended on patient preference, CBT‐I was effective even when patients preferred medication. This highlights the robustness of CBT‐I as a treatment option when preferences cannot be met. Similar findings have been observed for Sleep Restriction Therapy, a key component of CBT‐I, supporting its efficacy regardless of patient preference (Sidani et al. 2016). The effect of treatment preference was no longer significant when preference was assessed as a continuous acceptability variable, suggesting that treatment preference is relevant for outcome when it is more outspoken.

Our findings have significant clinical implications. Clinicians often assume elderly patients prefer pharmacological insomnia treatment, which hinders prescribing behavioural treatment methods (Dollman et al. 2003). Our findings suggest that this assumption is incorrect for patients with medical comorbidity. Additionally, if patients with severe insomnia prefer medication, this might be due to their belief that severe insomnia does not respond to CBT‐I. However, higher levels of insomnia severity have been found to predict better CBT‐I outcomes (Pruiksma et al. 2020). Offering alternative delivery formats for CBT‐I as well (e.g., internet based CBT‐I; van der Zweerde et al. 2020) might meet the needs of some patients for accessible treatment. Attributing psychological causes to insomnia was associated with a preference for CBT‐I, suggesting that improving understanding of these causes might shift preferences.

Our results highlight CBT‐I's robustness, as it is effective even when medication as AM is preferred. This supports its use as first‐line treatment, even when patients initially favour medication, particularly in cases where medication might not be the best option. Respecting patient preferences for psychological treatment like CBT‐I is important, as prescribing medication when not preferred can negatively influence treatment‐outcomes in a population with medical comorbidities. Although our study primarily compared CBT‐I with AM, it is probable that the outcomes would also be applicable to other medications, such as Benzodiazepine Receptor Agonists (BZRA) as zolpidem, given that the description we used of the effect of AM (“It has a calming effect and causes drowsiness”) aligns with those for BZRA's.

## 5 Strengths and Limitations

Strength of this study is that treatment preference was assessed both directly and indirectly and operationalised in two different ways. Furthermore, the study examined a broad range of possible predictors for treatment preference in a patient group with a wide spectrum of medical comorbidities. However, this study also has limitations. Expanding the sample to include participants who initially rejected the TIMELAPSE study may have increased variability in treatment preference factors. However, this was essential to make the sample more representative of the broader population with insomnia and medical comorbidities, aligning with the study's goal of inclusivity. The study sample consisted largely of TIMELAPSE participants and patients with a strong preference for CBT‐I were underrepresented as AM was only prescribed in the context of the TIMELAPSE study. This underrepresentation could also have led to an underrepresentation of individuals who might benefit more from CBT‐I, affecting the generalizability of the findings (King et al. 2005; Preference Collaborative Review Group 2008). Because of the selection bias, the finding that participants in our total sample preferred low‐dose AM above CBT‐I is not generalizable to a wider population of patients with insomnia and medical comorbidity.

Information about the severity of participants' medical conditions is lacking, although clinical factors like fatigue severity, impact of bodily pain on daytime functioning and physical functioning were present. Furthermore, although we attempted to describe the treatment methods in the TPP questionnaire as objectively as possible using guidelines of Witteman et al. (2015), the treatments do have distinctly different features. Participants therefore might have perceived the descriptions as unbalanced or biased. Another limitation is that the CAM‐I questionnaire has not been psychometrically validated.

Further research on treatment preference (and its relationship to treatment adherence and treatment outcome) in patients with insomnia and medical comorbidity outside the context of a RCT is warranted, including even a broader range of possible predictors of treatment preference such as self‐efficacy, previous experience with the treatment method, or perception of the perceived evidence base (Bluestein et al. 2011; Garland et al. 2018; Ibrahim and Sidani 2013).

## 6 Conclusion

Higher age and attributing psychological causes to insomnia predict a stronger preference for CBT‐I and higher insomnia severity, a stronger preference for low‐dose AM. Clinicians can recommend first‐line treatment CBT‐I even if the patient initially prefers medication, as CBT‐I remains effective. Respecting patient preferences for psychological treatment like CBT‐I is important, as prescribing medication when not preferred can negatively influence treatment outcomes in a population with medical comorbidities. Taking these factors into account in a clinical context facilitates targeted education and shared decision‐making with regard to insomnia treatment.

## Author Contributions

**Nynke Rauwerda:** writing – original draft, investigation, conceptualization, methodology, formal analysis, writing – review and editing, project administration. **Irene Pot:** conceptualization, investigation, writing – original draft, methodology, formal analysis, writing – review and editing. **Annemarie Braamse:** writing – review and editing. **Annemieke van Straten:** writing – review and editing. **Pythia van Nieuwkerk:** formal analysis, writing – review and editing, supervision. **Myrthe Boss:** writing – review and editing. **Marian Rikkert:** writing – review and editing. **Hans Knoop:** supervision, conceptualization, writing – review and editing, methodology.

## Ethics Statement

This study was approved by the Ethics Committee of Amsterdam University Medical Centre Research Ethics Committee (reference 2019_101) and the ethics committees of the participating hospitals.

## Consent

All participants provided informed consent. All authors gave the corresponding author consent to submit the manuscript for publication.

## Conflicts of Interest

The authors declare no conflicts of interest.

## Supporting information


**Data S1** Multivariate regression analyses of factors associated with insomnia treatment preference in the TIMELAPSE sample.

## Data Availability

Data supporting this study's findings can be obtained from the corresponding author upon reasonable request.

## Appendix A

Treatment descriptions of CBT‐I and low dose AM as used in the TPP, translated in English.

Cognitive behavioural therapy for Insomnia: Cognitive behavioral therapy for insomnia (CBT‐I) is a safe and effective form of treatment for long‐term insomnia. The goal of this treatment is to improve the sleep‐wake rhythm and improve sleep quality. CBT‐I consists of 7 group meetings of minimal 45 min each, supervised by a psychologist. The first 6 meetings take place weekly. After 6 weeks there is a follow‐up meeting. There is a maximum of 6 participants in each group. A workbook is used. Treatment components include sleep hygiene education, keeping a sleep diary, relaxation, changing thoughts and behaviors that disrupt sleep, and relapse prevention. An important part of CBT‐I is doing homework assignments. It takes time and effort to get used to new habits. Participants of CBT‐I may experience a temporary increase in fatigue and daytime sleepiness during the first weeks of treatment. There are no side effects or risks associated with the treatment.

Amitriptyline: Amitriptyline is a registered and safe antidepressant, most commonly used to treat depression or chronic pain. It has a calming effect and causes drowsiness. Practice has shown that the use of amitriptyline can improve sleep quality. That is why it is often prescribed as a ‘sleep aid’. Treatment with amitriptyline consists of a daily intake of 10 mg amitriptyline, shortly before going to sleep. If necessary, you can double the dose to 20 mg after 3 weeks. During the treatment you will visit the neurologist once and the sleep nurse once to discuss how you are sleeping. In general, patients with insomnia tolerate the low‐dose amitriptyline well. There are no major risks associated with the use of this medication. Sometimes users experience mild, reversible side effects such as blurred vision, nausea, sweating, dry mouth, constipation, dizziness and drowsiness. Usually these side effects subside or disappear completely after a few weeks. Some more serious side effects have been associated with a high dose of amitriptyline, but are not expected at the low dose of 10–20 mg per day.

AM: Amitriptyline; CBT‐I: Cognitive Behavioural Therapy for Insomnia; TPP: Treatment Perception and Preferences questionnaire.

## Appendix B

Prevalence (in numbers) of medical conditions (organised in ICD‐11 classification) of the total sample (*N* = 241).

**Table:**

Disease ICD‐11 classification	Total
Neoplasm	22
Diseases of the blood or blood‐forming organs	2
Diseases of the immune system	11
Endocrine, nutritional or metabolic diseases	71
Mental, behavioural or neurodevelopmental disorders	1
Sleep–wake disorders	36
Diseases of the nervous system	80
Diseases of the visual system	7
Diseases of the ear or mastoid process	9
Diseases of the circulatory system	30
Diseases of the respiratory system	33
Diseases of the digestive system	29
Diseases of the skin	6
Diseases of the musculoskeletal system and connective tissue	40
Diseases of the genitourinary system	3
Developmental anomalies	4
Symptoms, signs or clinical findings, not elsewhere classified	42
Injury, poisoning or certain other consequences of external causes	2
Factors influencing health status or contact with health services	1
Codes for special purposes: new diseases of uncertain aetiology and emergency use	5

Abbreviation: ICD‐11: International Classification of Diseases 11th Revision.

## References

[jsr70115-bib-0001] Aaronson, N. K. , M. Muller , P. D. Cohen , et al. 1998. “Translation, Validation, and Norming of the Dutch Language Version of the SF‐36 Health Survey in Community and Chronic Disease Populations.” Journal of Clinical Epidemiology 51, no. 11: 1055–1068. 9817123 10.1016/s0895-4356(98)00097-3

[jsr70115-bib-0002] American Psychiatric Association DSMTF . 2013. Diagnostic and Statistical Manual of Mental Disorders: DSM‐5. 5th ed. American Psychiatric Association.

[jsr70115-bib-0003] Bastien, C. H. , A. Vallieres , and C. M. Morin . 2001. “Validation of the Insomnia Severity Index as an Outcome Measure for Insomnia Research.” Sleep Medicine 2, no. 4: 297–307. 11438246 10.1016/s1389-9457(00)00065-4

[jsr70115-bib-0004] Bjelland, I. , A. A. Dahl , T. T. Haug , and D. Neckelmann . 2002. “The Validity of the Hospital Anxiety and Depression Scale. An Updated Literature Review.” Journal of Psychosomatic Research 52, no. 2: 69–77. 11832252 10.1016/s0022-3999(01)00296-3

[jsr70115-bib-0005] Bluestein, D. , A. C. Healey , and C. M. Rutledge . 2011. “Acceptability of Behavioral Treatments for Insomnia.” Journal of American Board of Family Medicine 24, no. 3: 272–280. 10.3122/jabfm.2011.03.100246 21551399

[jsr70115-bib-0006] Budhiraja, R. , T. Roth , D. W. Hudgel , P. Budhiraja , and C. L. Drake . 2011. “Prevalence and Polysomnographic Correlates of Insomnia Comorbid With Medical Disorders.” Sleep 34, no. 7: 859–867. 21731135 10.5665/SLEEP.1114 PMC3119827

[jsr70115-bib-0007] Buenaver, L. F. , D. Townsend , and J. C. Ong . 2019. “Delivering Cognitive Behavioral Therapy for Insomnia in the Real World: Considerations and Controversies.” Sleep Medicine Clinics 14, no. 2: 275–281. 31029193 10.1016/j.jsmc.2019.01.008

[jsr70115-bib-0008] Buysse, D. J. , S. Ancoli‐Israel , J. D. Edinger , K. L. Lichstein , and C. M. Morin . 2006. “Recommendations for a Standard Research Assessment of Insomnia.” Sleep 29, no. 9: 1155–1173. 17040003 10.1093/sleep/29.9.1155

[jsr70115-bib-0009] Carney, C. E. , J. D. Edinger , C. M. Morin , et al. 2010. “Examining Maladaptive Beliefs About Sleep Across Insomnia Patient Groups.” Journal of Psychosomatic Research 68, no. 1: 57–65. 20004301 10.1016/j.jpsychores.2009.08.007 PMC2796256

[jsr70115-bib-0010] Cheung, J. M. , D. J. Bartlett , C. L. Armour , and B. Saini . 2016. “Treating Insomnia: A Review of Patient Perceptions Toward Treatment.” Behavioral Sleep Medicine 14, no. 3: 235–266. 26240937 10.1080/15402002.2014.981818

[jsr70115-bib-0011] Cheung, J. M. Y. , D. J. Bartlett , C. L. Armour , B. Saini , and T. L. Laba . 2018. “Patient Preferences for Managing Insomnia: A Discrete Choice Experiment.” Patient 11, no. 5: 503–514. 29502237 10.1007/s40271-018-0303-y

[jsr70115-bib-0012] Cheung, J. M. Y. , X. W. Ji , and C. M. Morin . 2019. “Cognitive Behavioral Therapies for Insomnia and Hypnotic Medications: Considerations and Controversies.” Sleep Medicine Clinics 14, no. 2: 253–265. 31029191 10.1016/j.jsmc.2019.01.006

[jsr70115-bib-0013] Dikeos, D. , and G. Georgantopoulos . 2011. “Medical Comorbidity of Sleep Disorders.” Current Opinion in Psychiatry 24, no. 4: 346–354. 21587079 10.1097/YCO.0b013e3283473375

[jsr70115-bib-0014] Dollman, W. B. , V. T. LeBlanc , and E. E. Roughead . 2003. “Managing Insomnia in the Elderly—What Prevents Us Using Non‐Drug Options?” Journal of Clinical Pharmacy and Therapeutics 28, no. 6: 485–491. 14651672 10.1046/j.0269-4727.2003.00523.x

[jsr70115-bib-0015] Everitt, H. , D. S. Baldwin , B. Stuart , et al. 2018. “Antidepressants for Insomnia in Adults.” Cochrane Database of Systematic Reviews 5: CD010753. 29761479 10.1002/14651858.CD010753.pub2 PMC6494576

[jsr70115-bib-0016] Faul, F. , E. Erdfelder , A. Buchner , and A.‐G. Lang . 2009. “Statistical Power Analyses Using G* Power 3.1: Tests for Correlation and Regression Analyses.” Behavior Research Methods 41, no. 4: 1149–1160. 19897823 10.3758/BRM.41.4.1149

[jsr70115-bib-0017] Garland, S. N. , W. Eriksen , S. Song , et al. 2018. “Factors That Shape Preference for Acupuncture or Cognitive Behavioral Therapy for the Treatment of Insomnia in Cancer Patients.” Supportive Care in Cancer 26, no. 7: 2407–2415. 29423681 10.1007/s00520-018-4086-4 PMC6158018

[jsr70115-bib-0018] Harvey, A. G. , A. Soehner , T. Lombrozo , L. Belanger , J. Rifkin , and C. M. Morin . 2013. “'Folk Theories' About the Causes of Insomnia.” Cognitive Therapy and Research 37, no. 5: 1048–1057. 10.1007/s10608-013-9543-2 PMC3811969 24187398

[jsr70115-bib-0019] Hosmer, J. D. W. , S. Lemeshow , and R. X. Sturdivant . 2013. Applied Logistic Regression. John Wiley & Sons.

[jsr70115-bib-0020] Ibrahim, S. , and S. Sidani . 2013. “Preferences for Behavioral Therapies for Chronic Insomnia.” Health 5, no. 11: 1784–1790.

[jsr70115-bib-0021] Katz, D. A. , and C. A. McHorney . 1998. “Clinical Correlates of Insomnia in Patients With Chronic Illness.” Archives of Internal Medicine 158, no. 10: 1099–1107. 9605781 10.1001/archinte.158.10.1099

[jsr70115-bib-0022] King, M. , I. Nazareth , F. Lampe , et al. 2005. “Impact of Participant and Physician Intervention Preferences on Randomized Trials: A Systematic Review.” Journal of the American Medical Association 293, no. 9: 1089–1099. 15741531 10.1001/jama.293.9.1089

[jsr70115-bib-0023] Koffel, E. , A. D. Bramoweth , and C. S. Ulmer . 2018. “Increasing Access to and Utilization of Cognitive Behavioral Therapy for Insomnia (CBT‐I): A Narrative Review.” Journal of General Internal Medicine 33, no. 6: 955–962. 29619651 10.1007/s11606-018-4390-1 PMC5975165

[jsr70115-bib-0024] Morin, C. M. , G. Belleville , L. Belanger , and H. Ivers . 2011. “The Insomnia Severity Index: Psychometric Indicators to Detect Insomnia Cases and Evaluate Treatment Response.” Sleep 34, no. 5: 601–608. 21532953 10.1093/sleep/34.5.601 PMC3079939

[jsr70115-bib-0025] Morin, C. M. , B. Gaulier , T. Barry , and R. A. Kowatch . 1992. “Patients' Acceptance of Psychological and Pharmacological Therapies for Insomnia.” Sleep 15, no. 4: 302–305. 1519003 10.1093/sleep/15.4.302

[jsr70115-bib-0026] Morin, C. M. , A. Vallieres , and H. Ivers . 2007. “Dysfunctional Beliefs and Attitudes About Sleep (DBAS): Validation of a Brief Version (DBAS‐16).” Sleep 30, no. 11: 1547–1554. 18041487 10.1093/sleep/30.11.1547 PMC2082102

[jsr70115-bib-0027] Okajima, I. , and Y. Inoue . 2018. “Efficacy of Cognitive Behavioral Therapy for Comorbid Insomnia: A Meta‐Analysis.” Sleep and Biological Rhythms 16, no. 1: 21–35. 10.1007/s41105-022-00418-0 PMC10899977 38468616

[jsr70115-bib-0028] Omvik, S. , S. Pallesen , B. Bjorvatn , B. Sivertsen , O. E. Havik , and I. H. Nordhus . 2010. “Patient Characteristics and Predictors of Sleep Medication Use.” International Clinical Psychopharmacology 25, no. 2: 91–100. 20071997 10.1097/YIC.0b013e328334e5e6

[jsr70115-bib-0029] Perez, E. , E. K. Donovan , B. D. Rybarczyk , and J. M. Dzierzewski . 2022. “Insomnia Treatment Preferences Among Primary Care Patients.” Clinical Therapeutics 44, no. 4: 630–637. 35361532 10.1016/j.clinthera.2022.03.002 PMC9133067

[jsr70115-bib-0030] Preference Collaborative Review Group . 2008. “Patients' Preferences Within Randomised Trials: Systematic Review and Patient Level Meta‐Analysis.” BMJ 337: a1864. 18977792 10.1136/bmj.a1864 PMC2659956

[jsr70115-bib-0031] Pruiksma, K. E. , W. J. Hale , J. Mintz , et al. 2020. “Predictors of Cognitive Behavioral Therapy for Insomnia (CBTi) Outcomes in Active‐Duty U.S. Army Personnel.” Behavior Therapy 51, no. 4: 522–534. 32586427 10.1016/j.beth.2020.02.001

[jsr70115-bib-0032] Rauwerda, N. L. , H. Knoop , I. Pot , et al. 2021. “TIMELAPSE Study‐Efficacy of Low‐Dose Amitriptyline Versus Cognitive Behavioral Therapy for Chronic Insomnia in Patients With Medical Comorbidity: Study Protocol of a Randomized Controlled Multicenter Non‐Inferiority Trial.” Trials 22, no. 1: 904. 34895308 10.1186/s13063-021-05868-4 PMC8665718

[jsr70115-bib-0100] Rauwerda, N. , A. van Straten , A. Zondervan , et al. forthcoming. “Low Dose Amitriptyline Versus Cognitive Behavioral Therapy for Insomnia in Patients with Medical Comorbidity: Results of a Randomized Controlled Multicenter Non Inferiority Trial.” Sleeep. 10.1093/sleep/zsaf176 PMC12696364 40569003

[jsr70115-bib-0033] Ravyts, S. G. , E. Perez , and J. M. Dzierzewski . 2022. “Pain‐Related Beliefs About Sleep as a Predictor of Insomnia Symptoms and Treatment Acceptability.” Sleep Medicine 96: 122–127. 35640499 10.1016/j.sleep.2022.05.008 PMC9205612

[jsr70115-bib-0034] Razavi, D. , N. Delvaux , C. Farvacques , and E. Robaye . 1990. “Screening for Adjustment Disorders and Major Depressive Disorders in Cancer In‐Patients.” British Journal of Psychiatry 156: 79–83. 10.1192/bjp.156.1.79 2297623

[jsr70115-bib-0035] Riedel‐Heller, S. G. , H. Matschinger , and M. C. Angermeyer . 2005. “Mental Disorders—Who and What Might Help? Help‐Seeking and Treatment Preferences of the Lay Public.” Social Psychiatry and Psychiatric Epidemiology 40, no. 2: 167–174. 15685409 10.1007/s00127-005-0863-8

[jsr70115-bib-0036] Riemann, D. , C. A. Espie , E. Altena , et al. 2023. “The European Insomnia Guideline: An Update on the Diagnosis and Treatment of Insomnia 2023.” Journal of Sleep Research 32, no. 6: e14035. 38016484 10.1111/jsr.14035

[jsr70115-bib-0037] Riemann, D. , and M. L. Perlis . 2009. “The Treatments of Chronic Insomnia: A Review of Benzodiazepine Receptor Agonists and Psychological and Behavioral Therapies.” Sleep Medicine Reviews 13, no. 3: 205–214. 19201632 10.1016/j.smrv.2008.06.001

[jsr70115-bib-0038] Shaffer, K. M. , A. J. Applebaum , K. N. DuHamel , S. N. Garland , P. Gehrman , and J. J. Mao . 2020. “Cancer Survivors' Beliefs About the Causes of Their Insomnia: Associations of Causal Attributions With Survivor Characteristics.” Behavioral Sleep Medicine 18, no. 2: 177–189. 30475651 10.1080/15402002.2018.1546708 PMC6535375

[jsr70115-bib-0039] Sidani, S. , D. R. Epstein , M. Fox , and J. Miranda . 2018. “Psychometric Properties of the Treatment Perception and Preferences Measure.” Clinical Nursing Research 27, no. 6: 743–761. 27301566 10.1177/1054773816654137

[jsr70115-bib-0040] Sidani, S. , M. Fox , D. R. Epstein , and J. Miranda . 2016. “Challenges in Using the Randomized Trial Design to Examine the Influence of Treatment Preferences.” Canadian Journal of Nursing Research 48, no. 1: 7–13. 10.1177/0844562116665274 28841070

[jsr70115-bib-0041] Sidani, S. , J. Miranda , D. Epstein , and M. Fox . 2009. “Influence of Treatment Preferences on Validity: A Review.” Canadian Journal of Nursing Research 41, no. 4: 52–67. 20191713

[jsr70115-bib-0042] Sidani, S. , J. Miranda , D. R. Epstein , R. R. Bootzin , J. Cousins , and P. Moritz . 2009. “Relationships Between Personal Beliefs and Treatment Acceptability, and Preferences for Behavioral Treatments.” Behaviour Research and Therapy 47, no. 10: 823–829. 19604500 10.1016/j.brat.2009.06.009 PMC2742570

[jsr70115-bib-0043] Smith, M. T. , M. I. Huang , and R. Manber . 2005. “Cognitive Behavior Therapy for Chronic Insomnia Occurring Within the Context of Medical and Psychiatric Disorders.” Clinical Psychology Review 25, no. 5: 559–592. 15970367 10.1016/j.cpr.2005.04.004

[jsr70115-bib-0044] Spinhoven, P. , J. Ormel , P. P. Sloekers , G. I. Kempen , A. E. Speckens , and A. M. Van Hemert . 1997. “A Validation Study of the Hospital Anxiety and Depression Scale (HADS) in Different Groups of Dutch Subjects.” Psychological Medicine 27, no. 2: 363–370. 9089829 10.1017/s0033291796004382

[jsr70115-bib-0045] Taylor, D. J. , L. J. Mallory , K. L. Lichstein , H. H. Durrence , B. W. Riedel , and A. J. Bush . 2007. “Comorbidity of Chronic Insomnia With Medical Problems.” Sleep 30, no. 2: 213–218. 17326547 10.1093/sleep/30.2.213

[jsr70115-bib-0046] van der Zweerde, T. , J. Lancee , A. Ida Luik , and A. van Straten . 2020. “Internet‐Delivered Cognitive Behavioral Therapy for Insomnia: Tailoring Cognitive Behavioral Therapy for Insomnia for Patients With Chronic Insomnia.” Sleep Medicine Clinics 15, no. 2: 117–131. 32386688 10.1016/j.jsmc.2020.02.001

[jsr70115-bib-0047] Vincent, N. , and C. Lionberg . 2001. “Treatment Preference and Patient Satisfaction in Chronic Insomnia.” Sleep 24, no. 4: 411–417. 11403525 10.1093/sleep/24.4.411

[jsr70115-bib-0048] Witteman, H. O. , S. C. Dansokho , H. Colquhoun , et al. 2015. “User‐Centered Design and the Development of Patient Decision Aids: Protocol for a Systematic Review.” Systematic Reviews 4: 11. 25623074 10.1186/2046-4053-4-11 PMC4328638

[jsr70115-bib-0049] Worm‐Smeitink, M. , M. Gielissen , L. Bloot , et al. 2017. “The Assessment of Fatigue: Psychometric Qualities and Norms for the Checklist Individual Strength.” Journal of Psychosomatic Research 98: 40–46. 28554371 10.1016/j.jpsychores.2017.05.007

[jsr70115-bib-0050] Wu, J. Q. , E. R. Appleman , R. D. Salazar , and J. C. Ong . 2015. “Cognitive Behavioral Therapy for Insomnia Comorbid With Psychiatric and Medical Conditions: A Meta‐Analysis.” JAMA Internal Medicine 175, no. 9: 1461–1472. 26147487 10.1001/jamainternmed.2015.3006

