# Supplementary material for: Treatment Preferences in Patients With Insomnia and Medical Comorbidity: Associated Factors and Impact on Treatment‐Outcome
Source: J Sleep Res. 2025 Jun 30;35(1):e70115. doi: 10.1111/jsr.70115 (PMC12856101; doi:10.1111/jsr.70115)
Supplement: Supplementary file 1 — Data S1 Multivariate regression analyses of factors associated with insomnia treatment preference in the TIMELAPSE sample. [file JSR-35-e70115-s001.docx]

**Supplement 1.** Multivariate regression analyses of factors associated with insomnia treatment preference in the TIMELAPSE sample.

| *Model*** | *I* | | *II* | | *III* | | *IV* | | *V* | | *VI* | | *VII* | |
| --- | --- | --- | --- | --- | --- | --- | --- | --- | --- | --- | --- | --- | --- | --- |
| *Predictor* | *Stand. Β* | *p-value* | *Stand. Β* | *p-value* | *Stand. Β* | *p-value* | *Stand. Β* | *p-value* | *Stand. Β* | *p-value* | *Stand. Β* | *p-value* | *Stand. Β* | *p-value* |
| Current use of sleep medication | 0.11 | 0.17 | 0.11 | 0.17 | 0.10 | 0.18 | 0.11 | 0.16 | 0.10 | 0.18 | 0.13 | 0.09 | 0.12 | 0.11 |
| Psychological attribution | -0.16 | 0.04* | -0.16 | 0.04* | -0.16 | 0.04* | -0.15 | 0.05 | -0.15 | 0.06 | -0.11 | 0.14 |  |  |
| Dysfunctional beliefs and attitudes about sleep | 0.13 | 0.16 | 0.13 | 0.14 | 0.13 | 0.14 | 0.10 | 0.20 | 0.11 | 0.18 |  |  |  |  |
| Age | -0.12 | 0.13 | -0.12 | 0.13 | -0.10 | 0.17 | -0.09 | 0.22 |  |  |  |  |  |  |
| Fatigue severity | -0.10 | 0.32 | -0.10 | 0.30 | -0.06 | 0.45 |  |  |  |  |  |  |  |  |
| Physical functioning | -0.07 | 0.43 | -0.07 | 0.43 |  |  |  |  |  |  |  |  |  |  |
| Insomnia severity | 0.00 | 0.99 |  |  |  |  |  |  |  |  |  |  |  |  |
| Adj. R^2^ | 0.01 | 0.25 | 0.02 | 0.17 | 0.02 | 0.13 | 0.02 | 0.09 | 0.02 | 0.09 | 0.02 | 0.10 | 0.01 | 0.11 |
| R^2^ | 0.05 |  | 0.05 |  | 0.05 |  | 0.04 |  | 0.04 |  | 0.03 |  | 0.01 |  |
| N | 186 |  | 186 |  | 187 |  | 187 |  | 187 |  | 187 |  | 187 |  |

*p<0.05

** Model I included all candidate variables (p<0.25 in univariate regression analyses) of the total sample (TIMELAPSE and non-TIMELAPSE participants). In the subsequent models, non-significant variables (p > 0.05) were sequentially removed based on backward selection criteria.
